# Transcription Profiles Reveal Age-Dependent Variations of Photosynthetic Properties and Sugar Metabolism in Grape Leaves (*Vitis vinifera* L.)

**DOI:** 10.3390/ijms23042243

**Published:** 2022-02-17

**Authors:** You-Mei Li, Jia-Ling You, Wen-Feng Nie, Meng-Hao Sun, Zhao-Sen Xie

**Affiliations:** College of Horticulture and Plant Protection, Yangzhou University, Yangzhou 225000, China; 007426@yzu.edu.cn (Y.-M.L.); youjialing1948@163.com (J.-L.Y.); wfnie@yzu.edu.cn (W.-F.N.); mhsun163@163.com (M.-H.S.)

**Keywords:** grape, leaf, age, chlorophyll, photosynthesis, stomata, leaf ontogeny, sucrose

## Abstract

Leaves, considered as the ‘source’ organs, depend on the development stages because of the age-dependent photosynthesis and assimilation of leaves. However, the molecular mechanisms of age-dependent limitations on the function of leaves are seldom reported. In the present study, the photosynthesis-related characteristics and photoassimilates were investigated in grape leaves at six different age groups (Ll to L6) at micro-morphological, biochemical, and molecular levels. These results showed lower expression levels of genes associated with stomatal development, and chl biosynthesis resulted in fewer stomata and lowered chlorophyll a/b contents in L1 when compared to L3 and L5. The DEGs between L5 and L3/L1 were largely distributed at stomatal movement, carbon fixation, and sucrose and starch metabolism pathways, such as *STOMATAL ANION CHANNEL PROTEIN 1* (*SLAC1*), *FRUCTOSE-1,6-BISPHOSPHATE ALDOLASE* (*FBA1*), *SUCROSE-PHOSPHATE SYNTHASE* (*SPP1*), and *SUCROSE-PHOSPHATE PHOSPHATASE* (*SPS2*, *4*). These genes could be major candidate genes leading to increased photosynthesis capacity and sugar content in L5. The accumulation of starch grains in the chloroplast and palisade tissue of L5 and higher transcription levels of genes related to starch biosynthesis in L5 further supported the high ability of L5 to produce photoassimilates. Hence, our results provide insights for understanding different photosynthetic functions in age-dependent leaves in grape plants at the molecular level.

## 1. Introduction

In plants, mature leaves are considered as the ‘source’ organs that produce the photoassimilates, while fruits are the ‘sink’ organs that consume and/or accumulate resources from the ‘source’ during fruit development [1]. Providing sufficient carbohydrates for developing fruit is one of the essential factors to ensure fruit yield and quality. Several studies about sink formation and its genetic regulation have been reported to understand sugar metabolism and signaling in sink organs of fruit trees, including grapes [2]. However, the studies’ related photoassimilates produced in source organs are rarely reported, especially the age-dependent variations in photosynthetic assimilation of leaves.

Leaf age is related to photosynthetic productivity [3]. Mature leaves usually present higher photosynthetic capacity than young leaves [4]. The developing leaves initially import phloem-mobile nutrients from the rest of the plant; with maturation, leaves are capable of carbon fixation via photosynthesis and begin to export carbohydrates [5]. The light-saturated net photosynthetic rate on a leaf area basis peaks at or slightly before full leaf area expansion and then decreases with leaf senescence [6,7]. Carbon fixation is integrated over the entire growing season; thus, even small increases in the rate of photosynthesis can translate into yield increase [8]. Photosynthesis mainly occurs in leaves of land plants, which includes ‘light’ and ‘dark’ reactions [9]. Light reaction occurs with the absorption of light by pigments (chlorophyll and carotenoids) located in the thylakoid membrane, and then the light-driven electron transport reactions start with oxidizing water to oxygen and reducing the electron acceptor plastoquinone to plastoquinol by Photosystem II (PSII), which is a chlorophyll–protein complex embedded in the thylakoid membrane. Plastoquinol, in turn, carries the electrons derived from water to cytochrome *b*_6_*f* (cyt*b*_6_*f*), which is another thylakoid-embedded protein complex that oxidizes plastoquinol to plastoquinone and reduces a small water-soluble electron carrier protein plastocyanin. A second light reaction is then carried out by another chlorophyll protein complex called Photosystem I (PSI). PSI oxidizes plastocyanin and reduces another soluble electron carrier, protein ferredoxin, which resides in the stroma. Ferredoxin can then be used by the ferredoxin–NADP^+^ reductase (FNR) enzyme to reduce NADP^+^ to NADPH. These reactions are coupled to proton transfers that lead to the phosphorylation of adenosine diphosphate (ADP) into ATP [9]. Therefore, PSI, PSII, and cyt*b*_6_*f* are three major protein complexes involved in the light reaction [10]. Following the light reaction, dark reactions involve the fixation of CO_2_ into carbohydrate via the Calvin–Benson–Bassham (CBB) cycle that begins to occur in the stroma, being driven by NADPH and ATP. In summary, CO_2_ is first catalyzed by the enzyme ribulose-1,5-bisphosphate carboxylase/oxygenase (Rubisco) to combine with ribulose 1,5-bisphosphate [11] and form an unstable 6C intermediate that immediately splits into two molecules of 3-phosphoglycerate. 3-Phosphoglycerate is first phosphorylated by 3-phosphoglycerate kinase using ATP to form 1,3-bisphosphoglycerate. 1,3-Bisphosphoglycerate is then reduced by glyceraldehyde 3-phosphate dehydrogenase (GAPDH) using NADPH to form glyceraldehyde 3-phosphate (GAP, a triose or 3C sugar). GAP produced for the CBB cycle can be quickly converted by a range of metabolic pathways into amino acids, lipids, or sugars [9]. The GAPDH/chloroplast protein (CP12)/phosphoribulokinase (PRK) complexes play a central role in the regulation of the CBB cycle and are conserved in plants [12]. GAPDH and PRK positively regulate the CBB cycle in the light, but they will become inactivated when forming a complex with the oxidized CP12 in the dark [13]. Recently, many studies have demonstrated that the genetic modification of genes involved in the photosynthetic pathway, including cyt*b*_6_*f* complex component PetC, the Rubisco subunit, and ATP synthase, can change the maximum photosynthetic efficiency and biomass of plants [14,15]. The transcriptome landscapes of *Citrus sinensis* leaf indicated that DEGs related to PS I and II, ferredoxin, ATP synthase, cyt*b*_6_*f*, Rubisco, and GAPDH were predominantly upregulated in both transition and mature leaves compared with immature leaves. The same trend was found with glucose levels [16]. The photosynthetic capacity decreases with leaf senescence mainly because of the loss of Rubisco [7]. Therefore, photosynthesis in ‘source’ organs is one of the critical factors for carbon resources production [17]. 

A series of internal and external factors affects photosynthesis. Structurally, stomata control CO_2_ uptake from the ambient atmosphere for photosynthesis, determining plant productivity [18,19]. Most recently, a report claimed that higher stomatal density is beneficial for photosynthetic induction and biomass accumulation in Arabidopsis under fluctuating light [20]. In addition, stomatal size and responsiveness impact photosynthesis efficiency [19]. The genes responding for stomatal development and responsiveness have been reported: for example, *EPIDERMAL PATTERNING FACTOR* (*EPF*) family, *ERECTA-like kinase 1* (*ERL1*), and *TOO MANY MOUTHS* (*TMM*) enforce stomatal patterning [21,22]; the plasma membrane intrinsic proteins (PIPs) manage water to move through cell membranes and are involved in the regulation of stomatal movement in plants [23]; *STOMATAL ANION CHANNEL PROTEIN 1* (*SLAC1*) controls stomatal closure [24]. Apart from the stomatal factor, oxygenic photosynthesis needs chlorophyll (Chl) to absorb and transfer light energy to the two photosystems in which light conversion occurs [25]. Chl is one of the most abundant tetrapyrroles in land plants. Its synthesis begins from the precursor of 5-aminoleculinic acid (ALA) and contains a series of enzymatic steps as described in [26]. Chl a and Chl b are two different species of Chl produced in land plants. All the Chl b and most of Chl a function on light absorption and transport; only a few Chl a participate in light conversion [10]. In accordance with their function, Chl a is bound to different light-harvesting Chl-binding (LHC) proteins assembled both in the peripheral antenna complexes and the core antenna complexes of the PSI and II, while Chl b is specifically bound to the LHC proteins in peripheral antenna complexes [25]. 

The grapevine (*Vitis vinifera* L.) is a deciduous fruit tree that provides fresh fruit, dried raisins, and wine, with ecological and scientific value, and is cultivated world-wide. Sucrose produced from grape leaves is unloaded in the berry and metabolized as fructose and glucose; it is critical for berry growth and development [27]. Pervaiz et al. (2016) [28] have demonstrated that leaf growth and development alter photosynthesis and chlorophyll metabolic pathway in grapevine via transcriptomic analysis of grapevine leaves at the four developmental stages. Therefore, differently aged leaves could produce different sugar levels due to the difference in the photosynthesis metabolic pathway. Studying the age-dependent variations of sugar metabolism in grape leaves at a molecular level could reveal the function of sugar production and supply in differently aged leaves of fruit trees.

In the present study, the differences in photosynthetic properties (stomata attributes, chl content, photosynthetic parameters) and photosynthetic products (sucrose, glucose, and fructose levels) were investigated among differently aged leaves in ‘Pinot Noir’ grapevines. Additionally, RNA-Seq was conducted to analyze these differences at a molecular level. Combining with data from photosynthetic properties, photosynthetic products, and RNA-seq, we sought to: (1) investigate variations of photosynthetic properties and photosynthetic assimilation for differently aged leaves, (2) identify differentially expressed genes involved in photosynthesis-related traits and sugar metabolism, and (3) obtain candidate genes that most likely regulate the difference in photosynthetic assimilation across leaf developmental stages. The results provide more molecular insights to understand the difference of carbon assimilation among differently aged leaves and as such, provide a theoretical basis for improving the photosynthetic capacity and yield of grape trees. 

## 2. Results

### 2.1. Morphological Characteristics of Leaves with Different Ages

**Figure 1:**
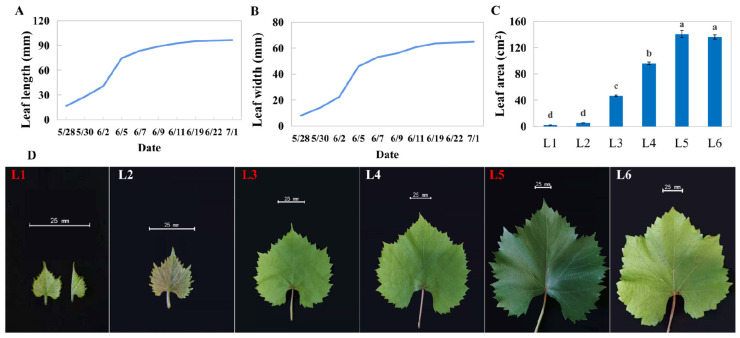
Leaf growth of ‘Pinot Noir’ grape. (**A**) Length of leaf midvein, (**B**) maximum width, (**C**) leaf area, and (**D**) photograph of leaf at different ages. The names with red font were the samples used to RNA-seq. L1 presents the newly undeveloped leaves; L2 presents initially opening leaves; L3 presents 1/3 * maximum leaf area of leaves; L4 presents 2/3 * maximum leaf area of leaves; L5 presents the leaves whose area no longer increases, and L6 presented leaves beginning to turn yellow. Different lowercase in subfigure C represented significant difference at 0.05 levels.

Sixty emerging undeveloped leaves from the top of uniform one-year shoots were marked as the first day of growth, and their sizes were measured periodically. The results showed that the length and width of these leaves increased dramatically in the first week; leaves grew slowly after one week and no longer significantly grew after about 30 days of growth (Figure 1A,B). We set the leaves no longer growing as L5 and collected the emerging and unexpanded leaves (L1), the newly expanded leaves (L2), the leaves with one-third the area of L5 (L3) and two-thirds the area of L5 (L4), and the aged leaves with yellow leaf edges (L6) for subsequent experiments (Figure 1D). In terms of appearance, differently aged leaves present differences in color and size (Figure 1C,D).

### 2.2. The Micro-Morphology of Stomata on Differently Aged Leaves 

**Figure 2:**
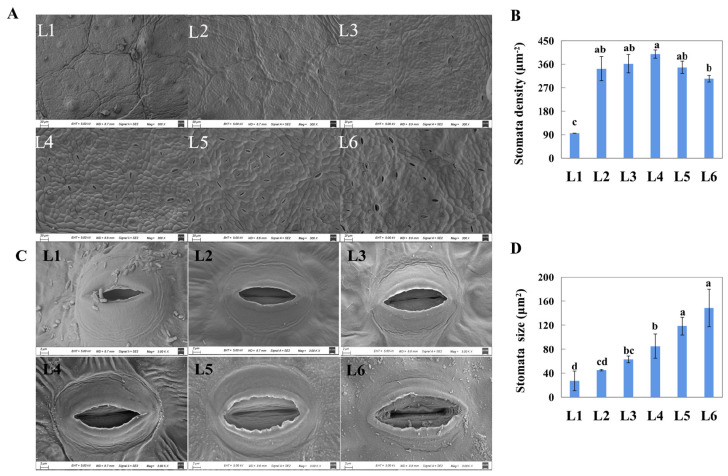
Scanning electron micrographs of stoma at differently aged leaves of ‘Pinot Noir’ grape. (**A**,**B**), stomatal density, scale: 20 μm. (**C**,**D**), stomatal shape and stomatal size, scale: 2 μm. Different lowercase in subfigure (**B**,**D**) represented significant difference at 0.05 levels.

The scanning electron microscopic images showed that the number of stomata in the unexpanded leaves (L1) was few, while the number was sharply increased in L2 leaves (Figure 2A). Consistently, L1 showed the lowest stomatal density (95 mm^−2^), while the others displayed stable density (303–398 mm^−2^, Figure 2B). With leaf growth, stomatal size increased (Figure 2C), and the sizes of L5 and L6 were obviously higher than those of the others (118.58 μm^2^ and 148.62 μm^2^, respectively, Figure 2D). 

### 2.3. The Physiological Difference of Leaves at Different Ages

**Figure 3:**
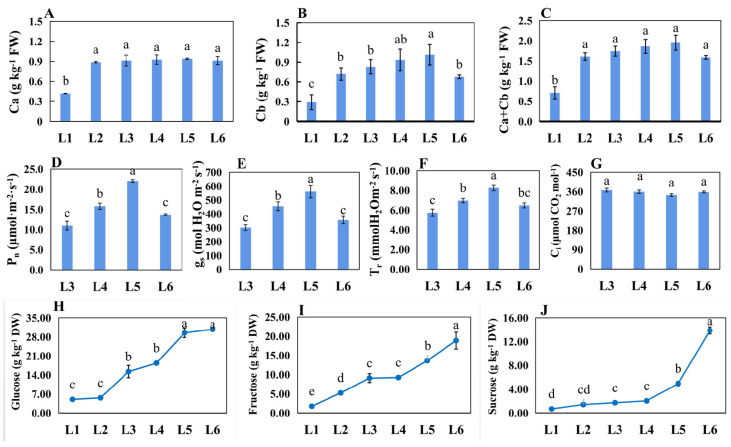
Physiology indexes related to photosynthesis analyzed among differently aged leaves of ‘Pinot Noir’ grape. (**A**–**C**) Chlorophyll content; (**D**–**G**) photosynthesis attributes, including net photosynthetic rate (*P*_n_), stomatal conductance (g_s_), transpiration rate (T_r_), and intercellular CO_2_ (C_i_); (**H**–**J**) sugar contents, including sucrose, fructose, and glucose. Bars indicate the mean ± standard error (*n* = 3). Different lowercase letters in Figure 3 indicate a significant difference at *p* < 0.05.

The Chl a and Chl b levels were significantly lower in L1 than in others. Additionally, the levels in L5 were the highest (Figure 3A–C). The values of *P*_n_, g_s_, and T_r_ were the highest in L5 but the lowest in L3. There were no differences in the C_i_ parameter among these leaves (Figure 3D–G). The contents of photosynthesis products, including sucrose, fructose, and glucose, were gradually increased from L1 to L6 (Figure 3H–J). Additionally, the increased extents of sucrose and fructose content were highest from the developmental transition from L5 to L6.

### 2.4. RNA-Seq Analysis and Functional Classification of DEGs

**Figure 4:**
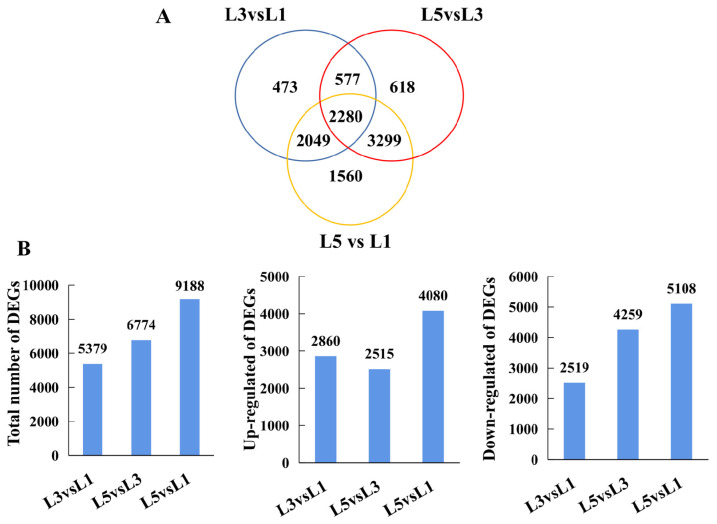
DEGs identified between L1, L3, and L5. Venn (**A**) and bar plot (**B**) illustrating the number of DEGs existing in L3 vs. L1, L5 vs. L3, and L5 vs. L1, respectively.

Nine cDNA libraries obtained 43198062, 42652276, 41550992, 43373766, 45964018, 42560230, 44057236, 44139002, and 43029030 clean reads filtered from raw data, respectively (Supplementary Table S1, see Supplementary Materials). A total of 10,856 non-redundant DEGs were identified via pairwise comparisons of L3 vs. L1, L5 vs. L3, and L3 vs. L1; the distribution of DEGs presented that 5379, 6774, and 9188 existed in L3 vs. L1, L5 vs. L3, and L3 vs. L1, respectively (Figure 4A, Supplementary Table S2). In the L3 vs. L1 comparison, 2860 were upregulated, and 2519 were downregulated; in the L5 vs. L3 comparison, 2515 were upregulated, and 4259 were downregulated; and in the L5 vs. L1 comparison, 4080 were upregulated and 5108 were downregulated (Figure 4B). The DEGs obtained from the L3 vs. L1, L5 vs. L3, and L5 vs. L1 comparisons were subjected to KEGG pathway enrichment analysis (Supplementary Table S3). Thirteen KEGG pathways were identified to be significantly enriched in the DEGs identified from the L3 vs. L1 comparison, including photosynthesis, carbon fixation in photosynthetic organisms, carotenoid biosynthesis, porphyrin and chlorophyll metabolism, and plant hormone signal transduction. Four KEGG pathways were significantly enriched in the L5 vs. L3 DEGs; the most significantly enriched KEGG was steroid biosynthesis, followed by amino sugar and nucleotide sugar metabolism, plant hormone signal transduction, and starch and sucrose metabolism. Lastly, twelve KEGG pathways were determined to be significantly enriched in the DEGs from the L5 vs. L1 comparison, including starch and sucrose metabolism, photosynthesis, carbon metabolism, and plant hormone signal transduction. The GO analysis also revealed that the DEGs belonged to several cellular components, molecular functions, and biological processes (Supplementary Table S4). In terms of biological processes, L3 and L5, in relation to L1, demonstrated changes in the expression level of genes encoding proteins involved in photosynthesis, light harvesting in photosystem I, the pigment metabolic/biosynthetic process, the carbohydrate biosynthetic process, and the polysaccharide metabolic process.

### 2.5. Expression Profile of the Genes Associated with Stomata Development and Movement in Differently Aged Leaves

The expression levels of genes responding to stomata patterns, including *EPF2*, *EPFL1*, *2*, *4*, *ERL1*, *EPFL9,* and *TMM*, were more highly expressed in L1 than L3 and L5 (Table 1). *SLAC1*, regulating stomatal closure, presented a higher expression level in L5 than that in L1. Seven plasma membrane intrinsic proteins (*PIPs*) engaged in stomata movements downregulated in L5, compared to L1 and L3. The MYB transcription factor (*MYB60*), regulating guard cell activity, showed significantly higher expression level in L5 and L3 than that in L1. Many genes involved in calcium signaling and chloride channel demonstrated significantly higher/lower expression levels in L5, compared to L1 and/or L3, including *CHLORIDE CHANNEL B* (*CLC-B*), *CLC-C*, *CLC-D*, and *CALMODULIN-LIKE* (*CML*). 

### 2.6. Chlorophyll-Related Genes in Differently Aged Leaves

**Figure 5:**
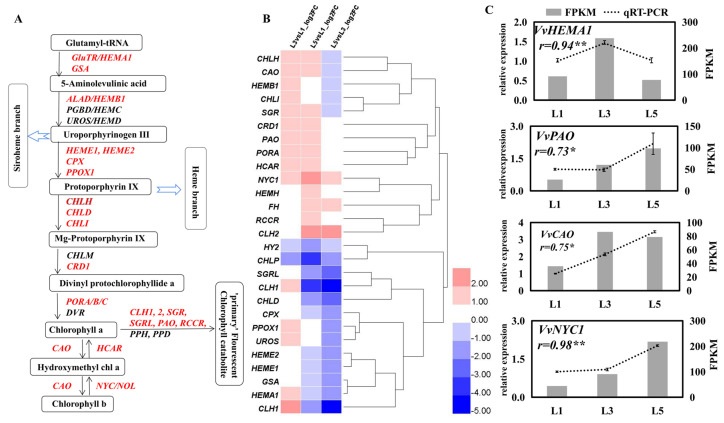
Expression profiles of chlorophyll-related genes in differently aged leaves of ‘Pinot Noir’ grape. (**A**) Chlorophyll biosynthesis and metabolism pathway. The genes marked with red font stand for the DEGs identified in present study; (**B**) heatmap of DEGs, Log2 (fold change) values were used for the heatmap; (**C**) identification by qRT-PCR of chlorophyll-related genes’ expression levels in leaves. The bar graph and line graph are derived from RNA-seq and qRT-PCR data, respectively. Values are means of three replicates ± SE. The asterisk presented the correlation coefficient (*r*) was significant at *p* < 0.05 (*) and *p* < 0.01 (**).

In total, 27 transcripts related to chlorophyll a/b related genes were significantly differentially expressed in L1, L3, and L5 leaves (Figure 5). *GluTR/HEMA1*, encoding the rate-limiting enzyme for the biosynthesis of 5-aminoleculinic acid (ALA), presented the highest expression level in L3 and no difference between L1 and L5 (Figure 5B,C). *GSA*, encoding the GSA aminotransferase, which catalyzes the transamination reaction to form ALA, kept constant expression levels between L1 and L3 and then declined in L5. A total of five genes involved in ALA dehydratase to protoporphyrin IX were found to have different expressions depending on leaf age. Three of them (*HEME1*, *HEME2*, and *CP6X*) displayed a similar expression pattern to the *GSA* gene (Figure 5B,C). *HEMB1* and *PPOX* were significantly upregulated in L3 compared to L1 and/or L5. Four genes responding for chlorophyll a biosynthesis (*CHLH*, *CHLI*, *CRD1*, and *PORA*) showed the highest expression levels but no significantly different expression level between L1 and L5 (Figure 5B,C). Three out of six chl-breakdown-related genes (*CLH1*, *SGR*, and *SGRL*) displayed the highest expression level in L3, while two (*CLH2* and *RCCR*) presented the highest in L5. *PAO*, opening the tetrapyrrole ring, which is a crucial step to chl degradation, was more highly expressed in L3 and L5 compared to L1 (Figure 5B). *CAO*, catalyzing the conversion of chl a to chl b, presented notably higher expression in L3 and L5 than that in L1. *NYC* and *HCAR*, responding to the first and second reaction of the Chl b to Chl a conversion, showed a higher expression level in L3 than that in L1 (Figure 5B,C). 

### 2.7. Expression Profiles of Photosynthesis-Related GENES in Differently Aged Leaves

**Figure 6:**
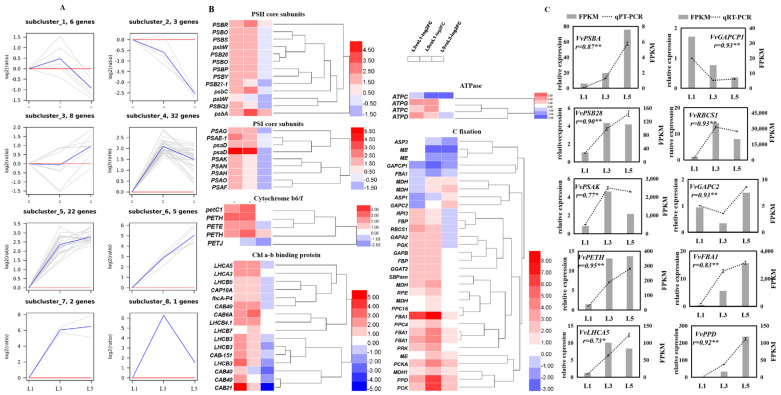
Expression profiles of photosynthesis-related genes in differently aged leaves of ‘Pinot Noir’ grape. (**A**) Hierarchical cluster analysis. FPKM values were used for the cluster analysis. (**B**) Heatmap of DEGs, log_2_ (fold change) values were used for the heatmap, (**C**) identification by qRT-PCR of some DEGs expression levels in leaves. The bar graph and line graph are derived from RNA-seq and qRT-PCR data, respectively. Values are means of three replicates ± SE. The asterisk presented the correlation coefficient (*r*) was significant at *p* < 0.05 (*) and *p* < 0.01 (**).

Expression profiles of photosynthesis- and carbon-fixation-related genes among differently aged leaves were analyzed by hierarchical clustering, which grouped these genes into eight subclusters (Figure 6A). Genes in subcluster four (32 genes) sharply increased from L1 to L3 and then declined from L3 to L5, while 22 genes in subcluster five remained constant or slightly increased between L3 and L5. These genes are involved in photosystem II, photosystem I, light harvest chlorophyll a-b binding protein, the cytochrome b6/f complex, and carbon fixation, such as *psbA*, *PSAK*, *LHCA5*, *petC1*, and *RBCS1*. Six genes in subcluster six showed a constantly sharp increase from L1 to L5; these genes responded for carbon fixation, including *PPD*, *PCK*, *PCKA*, and two *FBA1*. Subcluster one (six genes), subcluster two (three genes), and subcluster three (eight genes) presented a high expression level in L2, L1, and L3, respectively. *FBA1* and *psaD*, which are involved in carbon fixation and photosystem I, presented the subcluster seven expression profile. *CAB21*, which encoded light harvest chlorophyll a-b binding protein, was the only gene in subcluster eight. A heatmap shows the log_2_ (fold change) values of these over-presented DEGs in photosynthesis and carbon fixation pathways (Figure 6B). Most of them were significantly upregulated in L3 and L5 compared to L1, while remaining constant, increased, or declined between L5 and L3. qRT-PCR further detected ten DEGs, and their expression pattern accorded with RNA-seq data (Figure 6C). 

### 2.8. Expression Profiles of Sucrose and Starch Genes in Differently Aged Leaves

**Figure 7:**
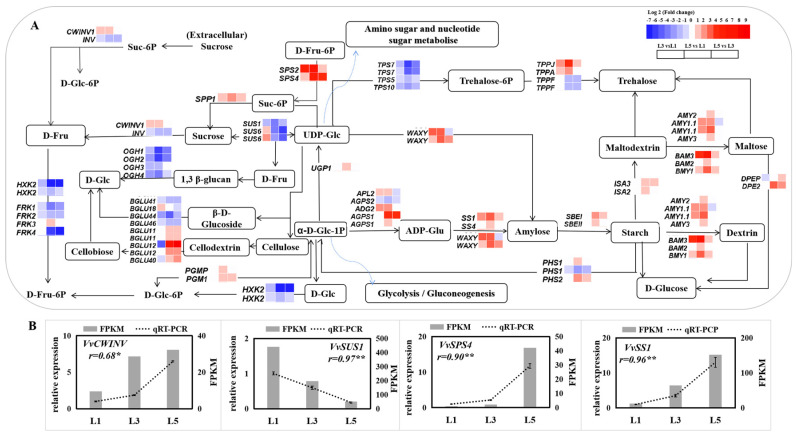
Expression of genes related to sucrose and starch metabolism in differently aged leaves of ‘Pinot Noir’ grape. (**A**) Pathway of sugar biosynthesis and metabolism and corresponding heat-map expression profiles of sugar-related DEGs. log_2_ (fold change) values were used for the heatmap. (**B**) Identification by qRT-PCR of DEGs expression levels in leaves. The bar graph and line graph are derived from RNA-seq and qRT-PCR data, respectively. Values are means of three replicates ± SE. The asterisk presented the correlation coefficient (*r*) was significant at *p* < 0.05 (*) and *p* < 0.01 (**).

Genes representing carbohydrate biosynthetic and metabolic transcriptional signatures in differently aged leaves are listed in Figure 7A. Of these, genes encoding sucrose-phosphatase 1 (SPP1), sucrose-phosphate synthase (SPS1, 2, 4), fructofuranosidase (CWINV), phosphoglucomutase (PGM1, PGMP), glucose-1-phosphate adenylyltransferase (AGPS1, ADG2, and APL2), starch synthase (SS1,4 and WAXY), alpha-amylase (AMY2, 3, 1.1), beta-amylase (BMY1, 2, 3), 1,4-alpha-glucan-branching enzyme (SBEI and SBEII), 4-alpha-glucanotransferase (DPEP and DPE2), and trehalose 6-phosphate phosphatase (TPPJ and TPPA) were significantly upregulated in L5, compared to L1 and/or L3, while genes encoding sucrose synthase (SUS1, 6), hexokinase (HKX2), trehalose-phosphate synthase (TPS5, 7, 10), and trehalose 6-phosphate phosphatase (TPPF) showed an opposite expression pattern. qRT-PCR detected eight of these sugar-related genes, and their expression pattern was consistent with RNA-seq data (Figure 7B).

## 3. Discussion

### 3.1. The Differences in Stomata Are Observed among Differently Aged Leaves at Morphology and Molecular Level 

Stomata are composed of guard cells and a microscopically small pore on the leaf surface that continuously balances CO_2_ supply for photosynthesis against water loss [18,19]. Studies have demonstrated that gs limits the photosynthetic rate under ambient CO_2_ concentration [24]. The g_s_ is affected by stomata density and stomata size [19]. In this study, the investigation of stomata-related attributes among differently aged leaves showed that the stomata densities were significantly higher in the fully expanded leaves (i.e., L2 to L6) than that of L1 leaves. The stomata size gradually increased with leaf aging (Figure 2). Therefore, these differences in stomatal attributes could explain the reason for the difference in g_s_ among these differently aged leaves (Figure 3). Transcriptome analysis showed that the expression levels of many genes involved in stomatal development were obviously differentially expressed among L1, L3, and L5 leaves (Table 1). Of these, *EPF2*, *EPFL4*, *EPFL6*, *ERL1*, and *TMM* have been reported to enforce stomatal patterning in many species [21,22]. More recently, a study demonstrated that *TMM* deletion blocks the negative regulation of stomatal development by *EPF1*,*2*-induced *ERf* signaling [29]. The declining expression of *TMM* from L1 to L5 leaves could restrict the transcription level of *EPF1/EPFLs* to maintain stomatal development. *EPFL9* has been demonstrated to be a positive regulator of stomata formation in Arabidopsis [30,31] in contrast to *EPF1* and *EPF2*. The transcript annotated as *EPFL9* was highly expressed in developing leaves (L1) but was almost not detected in mature leaves (L5) in the present study (Supplementary Table S2). This could suggest that stomatal patterning mainly occurs in developing leaves. Additionally, many genes have been reported to be involved in calcium sensors (calmodulin-like (*CML*)) and chloride channels (*ClC*) and are reported to be expressed in guard cells [32], demonstrating significantly higher/lower expression levels in L3 and/or L5 compared to L1 in the present study. Aquaporins such as the plasma membrane intrinsic proteins (PIPs) allow water to move through cell membranes and are vital for stomatal movement in plants [23,33]. *PIP2*-type aquaporins, which affect light-induced stomatal openings in Arabidopsis [33], were found to be differently expressed among L1, L3, and L5. *SLAC1*, encoding a stomatal anion channel and regulating stomatal closure in rice [24], was also significantly differently expressed in L3 and L5 compared to L1. VvMyb60, coding for a transcription factor involved in the regulation of guard cell activity and transpiration rate in grapevine [34], was also found in the present study, and its expression level is higher in L3 and L5 than of that in L1. Therefore, the upregulation or downregulation of these genes in L5 leaves could indicate a more active stomatal movement for L5, facilitating gas and water exchange between the leaf interior and the atmospheric environment.

### 3.2. Dynamic Regulation of Chl Biosynthesis and Metabolism-Related Genes Resulted in the Changed Chla/b Contents

Chla/b are key biochemical components in photosynthesis. A previous study has reported that photosynthesis increases are positively connected with increasing Chl a/b content [35]. In this study, no difference was observed in the Chl a levels among these differently aged leaves, except for undeveloped leaves (L1), while the difference in Chl b content was great, and the highest level was observed in L5 (Figure 3). Correspondingly, the *P*_n_ was the highest in L5 (Figure 3). At the molecular level, chlorophyll synthesis and metabolism genes were differently expressed in leaves of different ages (Figure 5). Glu-tRNA reductase is the rate-limiting enzyme for the biosynthesis of the tetrapyrrole precursor ALA, and three Arabidopsis orthology genes (*HEMA1*, *HEMA2*, and *HEMA3*) encode the GluTR isoforms. Since antisense *HEMA1*, Arabidopsis plants presented decreased levels of Chl and ALA; *HEMA1* is considered to play a major role in tetrapyrrole biosynthesis [36]. *HEMA2* and *HEMA3* are suggested to maybe have a limited physiological significance [37]. In the present study, *HEMA1* was found to have a higher expression level in L3 than that in L1 or in L5, which could be beneficial to ALA biosynthesis in L3. The first step of the Chl branch from ALA dehydratase is the Mg^2+^ inserting into protoporphyrin, a reaction processed by magnesium chelatase (MgCh) that is encoded by *CHLH*, *CHLI*, and *CHLD* in Arabidopsis [26]. Two orthology genes, *CHLH* and *CHLI*, were more highly expressed in L3 than in L1, and there was no significant difference between L3 and L5. *CRD1* and *PORA*, which were reported to be involved in subsequent steps of Chl a biosynthesis [26], showed the same expression pattern. Of these steps, the reduction in protochlorophyllide by POR is the first step in the overall greening processes in angiosperms [26]. Therefore, the upregulated expression of these genes in L3 and L5 could result in a higher Chl a content in L3 and L5 than that in L1, while no difference in their expression levels between L3 and L5 could be the main reason for the similar levels of Chl a between L3 and L5. Similarly, *CAO*, a gene catalyzing the conversion of Chl a to Chl b [38], was significantly upregulated in L3 compared with L1 but had no differential expression with L5. This could be the key gene leading to the change of Chl b content among L1, L3, and L5. Additionally, many Chl-breakdown-related genes were significantly expressed in L3 and/or L5 compared to L1, suggesting a major role for these genes in the chlorophyll cycle [39].

### 3.3. Genes Associated with Photosynthesis and Carbon Fixation Are Differentially Expressed among Differently Aged Leaves

Apart from stomatal factors, the genes encoding photosynthesis system components, such as PSI, PSII, LHCs, cyt*b*_6_*f*, and ATP synthase, also have a significant change among L1, L3, and L5 (Figure 6). Many studies have proven that photosynthesis has a positive relationship with the content of cyt*b*_6_*f* and Rubsico by using trans-genetic technology and physiological experiments [40,41]. One gene-encoding PetC protein of the cyt*b*_6_*f* complex [15,42] showed obviously higher expression levels in L3 and L5 compared to L1 in the present study. It has been reported to increase light conversion efficiency and photosynthesis in Arabidopsis (C3 plant) and Setaria viridis (C4 plant) when overexpressed [15,42]. Genes co-expressed with *PetC* displayed, in subcluster five, three other cytochrome b6/f complex components (two *PETH* and *PETE*), four light harvest chlorophyll a-b binding proteins (*LHCA5*, *LHCA3*, *CAB6A,* and *LHCB4.1*), one PSII subunit (*PSBR*), two PS subunits (*psbA* and *psbC*), one ATPase (*ATPC*), and ten carbon-fixation-related genes (*RBCS1*, *GAPB*, *SBPase*, *PGK*, *PRK*, *GGAT2*, *MDH*, *MDH1*, *RPE*, and *PPC4*). Of these, LHC proteins binding Chl a/b perform light-conversion functions [25]. SBPase was reported to increase photosynthesis rate, leaf area, and total biomass by as much as 30% in transgenic tobacco plants in high light when overexpressed [43]. *PRK* and *GAPB*, respectively, encoded phosphoribulokinase [44] and glyceraldehyde-3-phosphate dehydrogenase [45], two essential enzymes catalyzing the CBB cycle during photosynthesis. Rubisco is a rate-limiting enzyme that enables atmospheric carbon to convert into a biologically available carbon source during CBB [11]. Overexpression of Rubisco subunits with *RAF1* in maize increases Rubisco content and photosynthetic rate [41], while anti-Rubisco tobacco displayed opposite results [46]. In the present study, *RBCS1*, a Ribulose bisphosphate carboxylase small chain, which displayed higher transcription levels in L3 and L5 leaves, could be crucial to improve carbon assimilation in L3 and L5 leaves. Additionally, five carbon-fixation-related genes within subcluster six showed constantly increased expression levels with leaf age. *FBA1*, one of them in subcluster six, encodes fructose-1,6-bisphosphate aldolase and is similar to *FBPA* by Uematsu et al. [47], who reported that the overexpression of *FBPA* in transgenic tobacco plants resulted in increased photosynthesis and biomass at elevated levels of CO_2_. More recently, simultaneous overexpression of *SBPase*, *FBPA*, and cyanobacterial putative–inorganic carbon transporter B (*ictB*) in tobacco and populus increased the assimilation rate and biomass to a greater degree than in the wild type [48]. Collectively, many genes that have been demonstrated to promote the assimilation rate and biomass accumulation were significantly upregulated in L3 and/or L5, compared to L1, suggesting that the assimilation ability of differently aged leaves varies greatly. 

### 3.4. Sucrose- and Starch-Biosynthesis-Related Genes Were Involved in Differences in Sugar Contents of Differently Aged Leaves

The leaf is the main organ exporting photoassimilates into the sink for growth and development in plants such as fruit. Molecular mechanisms related to photoassimilates are involved in transportation, importation, and accumulation in sink organs [49]. However, limited information is available about the function of differently aged leaves on photoassimilates biosynthesis and metabolism. This study performed on grape leaves showed higher levels of sucrose, fructose, and glucose in mature leaves, L5 and L6, but lower levels in L1 and L2 (Figure 3). Three genes (*SPP1*, *SPS2*, and *SPS4*), identified and biochemically characterized as sucrose-phosphate synthase and sucrose-phosphate phosphatase in Arabidopsis, which catalyze the synthesis of Suc-6-P from UDP-glucose [50] and fructose-6-phosphate (Fru6P) and the irreversible hydrolysis of Suc-6-P to sucrose [51], respectively, were sharply upregulated in L5 in comparison to L1 and L3 (Figure 7), which is consistent with the increased sucrose content. Genes encoding cell wall apoplastic invertase (CWINV1) and 4-alpha-glucanotransferase (DPEP and DPE2), which are responsible for irreversibly hydrolyzing sucrose to fructose and glucose [52] and catalyzing maltose to glucose [53] in plant cells, respectively, presented higher expression levels in L3 and L5 than that in L1, while the fructokinase genes (*FRK1*, *2*, *4*), which catalyze fructose entering metabolism [54], obviously gradually declined in expression levels from L1 to L5. Therefore, these genes could play an important role in glucose and fructose accumulation during grape leaf development. Starch is basically a polymer of glucose, having glycoside linkages among glucose units [55]. It is regarded as a temporarily stored carbohydrate, as it can be converted into sucrose [56]. Many genes involved in starch biosynthesis and metabolism were significantly upregulated in L5 compared to L1 and L3, such as *AGPS1*, *SS1*, *SBEI*, *WAXY*, *AMY2*, *3*, *1.1,* and *BMY1*, *2*, *3*. The upregulation of a series of starch-biosynthesis-related genes in L5 could suggest that more hexoses could be converted to starch storage in mature leaves (L5) than in developing leaves (L1 and L3). More starch granules were observed in the chloroplast and palisade tissue of L5 leaves (Supplementary Figure S1) during our experiment. However, further studies need to study the mechanism of starch storage in mature leaves.

## 4. Materials and Methods

### 4.1. Plant Material and Sample Collection

This experiment was performed during 2020 at a vineyard at Yangzhou University, Yangzhou, Jiangsu Province, China (119°26′ E, 32°24′ N) using five-year-old ‘Pinot Noir’ grapevines. Sixty current-year shoots with uniform growth were selected, and the first undeveloped leaf at the top of current-year shoots was marked. The midvein length and maximum width of these leaves were measured periodically until the length and width no longer increased. Additionally, we used these marked leaves whose area no longer increased as the reference (L5), and the newly undeveloped leaves (L1), initially opening leaves (L2), leaves with 1/3 the maximum leaf area (L3), leaves with 2/3 the maximum leaf area (L4), and leaves beginning to turn yellow (L6) were collected between 9:00 and 10:00 a.m. Twenty leaves served as one biological replicate, and three replicates were frozen in liquid nitrogen and stored at −80 °C for RNA extraction. All secondary shoots from current-year shoots were removed in time during experiment. 

### 4.2. Leaf Area Measurement

Ten leaves from each replicate were scanned using UniscanA686 plane scanner (UNIS, Beijing, China), and the area was measured by ImageJ software fiji (Fiji Downloads (imagej.net)).

### 4.3. Photosynthesis Measurements

Ten leaves at each age were selected and labeled before measurement. The photosynthetic indexes including g_s_, net photosynthetic rate (*P*_n_), intercellular CO_2_ (C_i_), and transpiration rate (T_r_) of differently aged leaves were measured by a Li-6400 photosynthesis system (Li-COR, Lincoln, NE, USA), except for L1 and L2, whose sizes were too small to determine the photosynthetic parameters. The measurement was performed between 9:00 and 10:00 a.m. on a sunny day along with the following conditions: 1500 μmol m^−2^ s^−1^ light intensity, 380 μmol mol^−1^ CO_2_ concentration, 1.2–1.5 kPa vapor pressure deficit, 55–60% relative humidity, and 26–32 °C in the Li-COR-6400.

### 4.4. Scanning Electron Microscopy

Five leaves at six different ages were sampled, and three 5 * 5 mm^2^ squares were collected from the mid-lamina region of each leaf, avoiding areas in the vicinity of the midvein. The pre-treatment method for the electron microscopical observation of samples was described by Robinson et al. (1987). The epidermis abaxial surfaces were used to determine stomatal density and stomatal size by the GeminiSEM 300 field emission scanning electron microscope (Carl Zeiss Microscope GmbH, Oberkochen, Germany). The stomatal densities were recorded on five leaves of each age, based on counts at 100× magnification field. The length and width of the inner pores of five randomly selected stomata were measured at 3000× magnification field, and stomatal size was approximated as the product of length and width (μm^2^)

### 4.5. Determination of Chl a/b and Sugar Content

A total of 0.1 g of fresh leaves was used to extract Chl a/b content via the method reported by Burnison [57]. A total of 0.5 g of dry leaves was used to extract sugars for high-performance liquid chromatography (HPLC) analysis. The methods were described by Barros et al. [58], with an extra step to remove the pigment using the Agilent Sample Prep Solutions (Agilent Technologies Inc., Palo Alto, CA, USA) before HPLC analysis.

### 4.6. Total RNA Extraction

Leaves from L1, L3, and L5 were used for RNA-seq analysis. Three biological replicates were performed. Total RNA was extracted using the spectrumTM Plant Total RNA kit (Sigma-Aldrich, St. Louis, MO, USA). RNA integrity was detected on 1% agarose gels as well as in a Bioanalyzer 2100 System (Agilent Technologies, CA, USA), RNA purity was assessed using the NanoPhotometer^®^ spectrophotometer (Implen, Westlake Village, CA, USA), and RNA concentration was verified according to a Qubit RNA Assay Kit (Life Technologies, Carlsbad, CA, USA). RNA-seq analysis was carried out based on the RNA passed the quality tests.

### 4.7. RNA-Seq and Analysis

Nine RNA samples were used for sequence based on the Illumina Hiseq 4000 (San Diego, CA, USA) platform in the Novogene Institute (Novogene, Tianjin, China). Raw reads were first subjected to quality control and then processed to obtain clean reads with in-house Perl scripts. After removing the sequence with adapters, a sequence comprising more than 10% unknown bases, and a low-quality sequence, the clean reads were mapped to the Vitis vinifera reference genome [59] by TopHat v2.0.6 [60]. The clean reads were normalized into fragments per kilobase of transcript per million mapped read (FPKM) values to determine the expression level of each gene according to Trapnell et al. [61].

### 4.8. Identification and Analysis of Differentially Expressed Genes (DEGs)

DEGs were identified using the DESeq R package [62]. Significant DEGs were determined with an adjusted *p* value (*p*adj) of <0.05 and |log_2_ (fold change)| ≥ 1. The overlapping DEGs were presented in VennDiagram (Draw Venn Diagram.ugent.be/webtools/Venn/ (accessed on 11 January 2022)). topGO 2.18.0 [63] and clusterProfile 3.10.1 [64] were used to analyze the statistical enrichment of DEGs in gene-ontology (GO) and Kyoto Encyclopedia of Genes and Genomes (KEGG) pathway, respectively. GO and KEGG terms with an adjusted *p* value of ≤0.05 were considered significantly enriched. The hierarchical clustering analysis was performed with the H-cluster R package.

### 4.9. Quantitative Real-Time Polymerase Chain Reaction (RT-qPCR) Analysis

Total RNA was extracted from independent leaves collected at the same developmental stages as those used in the RNA-seq. First-strand cDNA was obtained using PrimeScriptTM RT reagent Kit with gDNA Eraser (Perfect Real Time) (TaKaRa, Dalian, China). The primers for RT-qPCR were designed with Primer 6.0 and are listed in Supplementary Table S5. RT-qPCR was performed on the CFX connect Real Time PCR Detection System (Bio-Rad) using SYBR Premix ExTaqTM II (Tli RNaseH Plus) (Takara, Beijing, China). The RT-qPCR protocol was processed based on the manufacture of SYBR Premix ExTaqTM II kit. *ACTIN* was used as internal reference control. The relative expression of detected genes was calculated with the 2^−ΔΔCt^ method [65].

### 4.10. Statistical Analysis

Stomata index, Chl a/b, photosynthesis attributes, and sugar content were subjected to variance analysis. Tukey’s test was used for calculation of means at *p* < 0.05.

## 5. Conclusions

In this study, grape leaves at six different developmental stages with different photosynthesis-related attributes, including stomatal morphology, chlorophyll content, and photosynthetic products content, as well as transcription profiling, were fully calculated. Morphological, physiological, and molecular data analyses revealed that different expressions of genes associated with stomatal development and chlorophyll a biosynthesis resulted in the difference of stomatal density and chlorophyll content between L1 and L3, respectively, which are the critical factors that affect photosynthesis and carbon fixation in photosynthetic organisms, while the differences between L5 and L3 were mainly manifested in stomatal opening size and sugar content. The expression pattern of genes related to stomatal movement, carbon fixation, and starch and sucrose metabolism were consistent with the change in stomatal opening size and sugar content. These results provide insights into the difference of photosynthesis assimilation among differently aged leaves in grapes, which could provide foundation data for subsequent studies on source–sink relationships in grapes.

## Figures and Tables

**Table 1:** The different expression genes associated with stomata pattern and movement among differently aged leaves.

Classification	Gene ID	Gene Name	log2^FC(L3vsL1)^	log2^FC(L5vsL1)^	log2^FC(L5vsL3)^	*p*adj^L3vsL1^	*p*adj^L5vsL1^	*p*adj^L5vsL3^
Stomata pattern	VIT_05s0062g01380	*EPIDERMAL PATTERNING FACTOR LIKE9* (*EPFL9*)	−1.32	−4.17	−2.90	4.35 × 10^−1^	3.63 × 10^−1^	6.29 × 10^−2^
	VIT_18s0164g00010	*EPIDERMAL PATTERNING FACTOR2* (*EPF2*)	−2.92	−9.76	−6.88	2.32 × 10^−23^	2.41 × 10^−15^	7.37 × 10^−7^
	VIT_18s0001g08350	*EPIDERMAL PATTERNING FACTOR LIKE2* (*EPFL2*)	−5.79	−6.66	−0.95	5.64 × 10^−7^	4.33 × 10^−7^	7.47 × 10^−1^
	VIT_06s0004g07880	*EPIDERMAL PATTERNING FACTOR LIKE6* (*EPFL6*)	−2.68	−1.26	1.38	9.31 × 10^−36^	1.19 × 10^−10^	3.18 × 10^−7^
	VIT_05s0020g04050	*EPIDERMAL PATTERNING FACTOR LIKE4* (*EPFL4*)	−6.17	−8.99	−2.95	2.17 × 10^−15^	1.35 × 10^−13^	3.38 × 10^−1^
	VIT_00s0391g00030	*EPIDERMAL PATTERNING FACTOR LIKE1* (*EPFL1*)	−2.07	−3.64	−1.61	1.07 × 10^−11^	2.90 × 10^−18^	2.12 × 10^−3^
	VIT_00s0386g00040	*EPIDERMAL PATTERNING FACTOR LIKE2* (*EPFL2*)	−1.52	−1.49	−0.02	1.09 × 10^−6^	1.37 × 10^−6^	9.54 × 10^−1^
	VIT_09s0002g07030	*TOO MANY MOUTHS* (*TMM*)	−5.67	−11.02	−5.40	7.77 × 10^−55^	3.53 × 10^−20^	4.66 × 10^−4^
	VIT_16s0022g02030	*ERECTA-like kinase 1* (*ERL1*)	−2.91	−5.93	−3.06	1.96 × 10^−58^	4.28 × 10^−117^	1.95 × 10^−17^
Stomata movement	VIT_02s0025g04930	*STOMATAL ANION CHANNEL PROTEIN 1* (*SLAC1*)	3.07	3.77	0.65	4.33 × 10^−28^	3.22 × 10^−41^	2.47 × 10^−4^
	VIT_01s0010g02940	*CALMODULIN-LIKE31* (*CML31*)	−4.29	−2.67	1.58	2.13 × 10^−3^	3.12 × 10^−2^	2.68 × 10^−1^
	VIT_18s0122g00180	*CALMODULIN-LIKE37* (*CML37*)	−3.29	−1.97	1.27	4.78 × 10^−4^	5.35 × 10^−2^	2.39 × 10^−3^
	VIT_18s0001g11830	*CALMODULIN-LIKE41* (*CML41*)	−0.69	1.59	2.24	3.64 × 10^−3^	5.45 × 10^−8^	1.02 × 10^−17^
	VIT_18s0001g03880	*CALMODULIN-LIKE29* (*CML29*)	6.49	8.58	2.04	4.50 × 10^−76^	1.24 × 10^−226^	5.25 × 10^−23^
	VIT_18s0001g01630	*CALMODULIN-LIKE44* (*CML44*)	5.63	9.25	3.58	3.88 × 10^−195^	7.97 × 10^−293^	1.02 × 10^−91^
	VIT_17s0000g01630	*CALMODULIN-LIKE19* (*CML19*)	0.06	−1.70	−1.80	8.32 × 10^−1^	2.01 × 10^−8^	5.19 × 10^−13^
	VIT_16s0039g01880	*CALMODULIN-LIKE18* (*CML18*)	0.20	1.28	1.04	6.22 × 10^−1^	2.80 × 10^−3^	1.45 × 10^−2^
	VIT_14s0030g02150	*CALMODULIN-LIKE1* (*CML11*)	−0.71	3.39	4.09	6.45 × 10^−1^	6.46 × 10^−6^	2.41 × 10^−6^
	VIT_11s0016g05740	*CALMODULIN-LIKE5* (*CML5*)	1.23	1.25	−0.03	1.02 × 10^−12^	6.04 × 10^−13^	8.69 × 10^−1^
	VIT_03s0063g00530	*CALMODULIN-LIKE30* (*CML30*)	1.50	3.11	1.56	8.51 × 10^−8^	3.62 × 10^−26^	1.27 × 10^−10^
	VIT_14s0068g02190	*CHLORIDE CHANNEL B* (*CLC-B*)	−0.38	−2.37	−2.04	8.09 × 10^−2^	1.14 × 10^−11^	6.02 × 10^−10^
	VIT_07s0130g00400	*CHLORIDE CHANNEL C* (*CLC-C*)	1.41	1.44	−0.02	2.63 × 10^−16^	2.03 × 10^−18^	8.68 × 10^−1^
	VIT_03s0038g04260	*CHLORIDE CHANNEL B* (*CLC-E*)	0.98	1.76	0.73	1.56 × 10^−14^	5.44 × 10^−33^	2.67 × 10^−8^
	VIT_08s0040g01890	*PLASMA MEMBRANE INTRINSIC PROTEIN2-1* (*PIP2-1*)	−0.63	−5.08	−4.50	3.75 × 10^−4^	1.32 × 10^−11^	1.79 × 10^−9^
	VIT_06s0004g02850	*PLASMA MEMBRANE INTRINSIC PROTEINS2-7* (*PIP2-7*)	−0.18	−2.45	−2.32	3.92 × 10^−1^	4.52 × 10^−17^	1.34 × 10^−24^
	VIT_03s0038g02520	*PLASMA MEMBRANE INTRINSIC PROTEINS2-7* (*PIP2-7*)	−0.75	−2.00	−1.30	1.25 × 10^−9^	1.49 × 10^−26^	1.81 × 10^−11^
	VIT_03s0038g01410	*PLASMA MEMBRANE INTRINSIC PROTEINS1-3* (*PIP1-3*)	4.09	1.46	−2.67	1.40 × 10^−110^	7.08 × 10^−11^	9.78 × 10^−48^
	VIT_15s0046g02420	*PLASMA MEMBRANE INTRINSIC PROTEINS1-1* (*PIP1-1*)	−0.43	−1.71	−1.33	1.47 × 10^−2^	3.29 × 10^−19^	2.09 × 10^−18^
	VIT_15s0046g02410	*PLASMA MEMBRANE INTRINSIC PROTEINS1-2* (*PIP1-2*)	−1.06	−4.40	−3.38	3.61 × 10^−9^	2.70 × 10^−94^	5.84 × 10^−46^
	VIT_13s0067g00220	*PLASMA MEMBRANE INTRINSIC PROTEINS1-2* (*PIP1-2*)	−0.07	−2.88	−2.86	6.54 × 10^−1^	6.33 × 10^−4^	6.21 × 10^−4^
	VIT_08s0056g00800	*MYB transcription factor 60* (*MYB60*)	2.87	2.91	0.00	1.39 × 10^−38^	3.20 × 10^−39^	9.99 × 10^−1^

Note: The blue means that the expression level is down-regulated, the red means the gene expression level is up-regulated. The darker color presented the higher level of up/down-regulation.

## Data Availability

Datasets supporting the conclusions of this article are included within the article and its additional files.

## Supplementary Materials

The following supporting information can be downloaded at: https://www.mdpi.com/article/10.3390/ijms23042243/s1.
