# Supplementary material for: Transcription Profiles Reveal Age-Dependent Variations of Photosynthetic Properties and Sugar Metabolism in Grape Leaves (Vitis vinifera L.)
Source: Int J Mol Sci. 2022 Feb 17;23(4):2243. doi: 10.3390/ijms23042243 (PMC8876361; doi:10.3390/ijms23042243)
Supplement: Supplementary file 1 [file ijms-23-02243-s001.zip › ijms-1573761-supplementary/Suppmentary Figures1,2.pdf]

1. Supplementary Figures

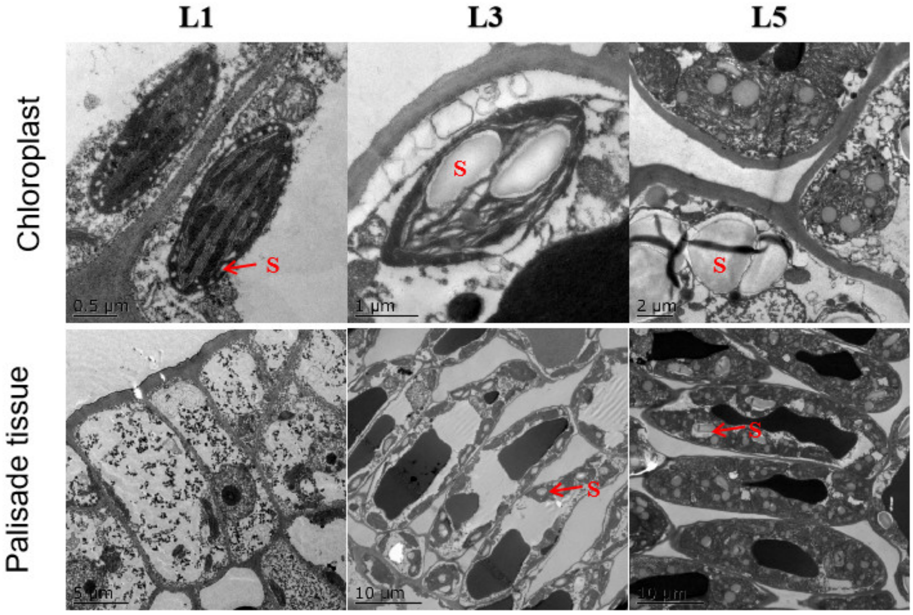

Figure S1. Morphological characteristics of leaf anatomical structure at different age leaves observed via Tecnai 12 transmission electron microscopy (TEM) in 'pinot Noir' grapevine. S means starch grains.
